# The effect of intrathecal pethidine on post-spinal anesthesia shivering after cesarean section: a systematic review and meta-analysis

**DOI:** 10.1097/MS9.0000000000002354

**Published:** 2024-07-22

**Authors:** Muhammad Afzal, Amber Lee, Muhammad Asad, Alya Ali, Ameer Mustafa Farrukh, Bader Semakieh, Yaxel Levin-Carrion, Shah Rukh Shah, Qaisar Ali Khan

**Affiliations:** a St. George’s University School of Medicine, True Blue, Grenada; b Arkansas College of Osteopathic Medicine, Fort Smith, AR; c Nassau University Medical Center Long Island, East Meadow, NY; d Rutgers New Jersey Medical School, Newark, NJ, USA; e Lady Reading Hospital; f Khyber Teaching Hospital MTI KTH, Peshawar; g Khyber Medical University Institute of Medical Sciences, Kohat, Pakistan; h University of Galway School of Medicine, Galway, Ireland

**Keywords:** pethidine, spinal anesthesia, cesarean section, surgical complications

## Abstract

**Background:**

Spinal anesthesia is the most preferred method for cesarean section (C-section). This meta-analysis was performed to determine the effect of low and high intrathecal doses of pethidine on the maternal outcomes after C-section.

**Methods:**

A systematic search of PubMed, Scopus, Cochrane Library, and Google Scholar was performed. Random-effects meta-analysis was performed to derive odds ratios (ORs) from dichotomous data.

**Results:**

Seventeen randomized controlled trials with 1304 C-section patients were included. Patients who had received intrathecal pethidine experienced decreased shivering and intensity of shivering (OR 0.13; *P*<0.001) and (OR 0.21; *P*<0.001), respectively. Moreover, vomiting (OR 2.47; *P*=0.002) and pruritus (OR 5.92; *P*<0.001) were significantly higher in the pethidine group. There was no statistically significant difference in the incidence of nausea (OR 2.55; *P*=0.06) and hypotension (OR 0.91; *P*=0.67).

**Conclusions:**

Intrathecal pethidine can effectively decrease shivering, although it increases the risk of vomiting and pruritus. No significant difference was found both in the maternal hypotension and nausea.

## Introduction

Highlights

- Pethidine is a synthetic phenylpiperidine derivative opioid that acts on μ and κ opioid receptors. Intravenous (IV) pethidine has long been used to treat and prevent shivering during surgery and the emergence from anesthesia. The anti-shivering mechanism is activated by IV pethidine acting on kappa-opioid receptors.
- Unlike IV pethidine, the advantages and disadvantages of intrathecal pethidine are unclear.
- Intrathecal pethidine effectively reduces the severity and incidence of post-spinal anesthesia shivering.
- Low doses increase pruritus, nausea, and vomiting while showing no association with hypotension.
- Surgeons could consider administering intrathecal pethidine at appropriate doses to subside PSAS effectively. However, continuous monitoring is needed to manage the side effects.

Spinal anesthesia is the most used anesthesia for elective and emergency cesarean section (CS) scenarios due to its simplicity and effective performance, low cost and quick application of anesthesia, providing sufficient analgesia and muscle relaxation for the procedure^1,2^. It is suggested by the Royal College of Obstetricians and Gynecologists that CS should be performed “with an urgency appropriate to the risk to the baby and the safety of the mother” as the target decision to delivery interval is achieved^3^. Anesthetic goals during CS include a specific anesthetic level to optimize surgical conditions and reduce maternal recall; adequate oxygenation of the mother and fetus; and minimal transfer of anesthesia to fetus^4^. CS is likely to have several adverse effects on the mother and the baby. For instance, mothers experience pain post- C-section likely due to spinal anesthesia wearing off without additional analgesia given^5^. Additionally, mothers experience hypotension, which is the most common minor postoperative complication of spinal anesthesia. Maternal hypotension is associated with bouts of nausea and vomiting with a risk of fetal acidosis. Shivering is another postoperative complication that can be troublesome as it interferes with the comfort and monitoring of the mother. Shivering results in increased oxygen consumption and carbon dioxide production, especially in mothers with low (thermoregulatory shivering) and high core temperatures (non-thermoregulatory shivering)^6,7^.

Pethidine or Meperidine is a synthetic phenylpiperidine derivative opioid that acts on μ and *k* opioid receptors. For a long period of time, intravenous (IV) pethidine has been used for the treatment and prevention of shivering during surgery and emergence. The anti-shivering mechanism is activated by IV pethidine acting on kappa-opioid receptors. Unlike IV pethidine, advantages and disadvantages of intrathecal pethidine are unclear^8,9^. For this purpose, we conducted this systematic review and meta-analysis to determine the efficacy of low-dose and high-dose intrathecal pethidine on CS patients with spinal anesthesia.

## Methods

This systematic review and meta-analysis was performed in accordance with the Preferred Reporting Items for Systematic Review and Meta-Analysis (PRISMA, Supplemental Digital Content 1, http://links.lww.com/MS9/A563)^10^ and the framework laid out by the Cochrane Collaboration^11^.

### Literature search and study selection

A comprehensive literature search of electronic databases, PubMed, Scopus, and Cochrane Library was performed from inception till September 2023. The following keywords were used in the search string: “meperidine”, “pethidine”, “intrathecal”, “spinal anesthesia”, and “cesarean section”. Detailed search strategy used in each database is shown in supplementary material Table S1, Supplemental Digital Content 2, http://links.lww.com/MS9/A564.

Articles initially shortlisted from each database were exported to Endnote Reference Library (Version X7.5; Clarivate Analytics, Philadelphia, Pennsylvania) software, where deduplication was performed. Two reviewers thoroughly reviewed the remaining articles first by title, then abstract, and finally a full-text evaluation was performed based on a preset eligibility criterion. Disagreements were resolved by consensus. The following inclusion criteria was employed to shortlist studies (1): randomized controlled trials (RCTs) (2), females undergoing cesarean section with spinal anesthesia (3), intrathecal meperidine (pethidine) administration between 5 and 50 mg (4), compared with bupivacaine or normal saline (5), studies involving the measurement of shivering or intensity of shivering. No language restrictions were placed when shortlisting articles. Exclusion criteria were the use of local anesthesia, non-elective surgery, combination of opioids in intervention or control, studies of observational nature, and studies whose full text was unavailable.

### Data extraction and quality assessment

Two independent reviewers conducted the data extraction. Any disagreement was resolved by discussion. Additionally, a snowball searching method was applied where articles were found by going through the citations of relevant articles. Data extraction was performed of the following characteristics: study design, country of study, number of patients in each group, dosage of pethidine, and primary and secondary endpoints. In this study incidence and severity of shivering were the primary outcomes. Adverse events including pruritus, nausea, vomiting, and hypotension were the secondary outcomes. Quality assessment was performed using the Risk of Bias-2 (RoB-2) tool of the Cochrane Collaboration for randomized controlled trials^12^. Differences between the two independent reviewers in quality assessment were resolved by discussion. We performed an evaluation of our meta-analysis using the AMSTAR-2 checklist, Supplemental Digital Content 3, http://links.lww.com/MS9/A565 ^13^. Our meta-analysis was found to be of high-quality.

### Statistical analysis

Review Manager (version 5.4.1; Copenhagen: The Nordic Cochrane Centre, The Cochrane Collaboration, 2020) and Comprehensive Meta-Analysis were used for data analysis. A random-effects model was employed to derive odds ratios (ORs) and corresponding 95% CIs. Publication bias was assessed by generating funnel plots and Egger’s regression test (0.00001). A *P* value less than 0.05 was considered statistically significant for all outcomes. Heterogeneity was assessed with Higgin’s I^2^ test. A value of I^2^=25–50% was considered mild, 50–75% as moderate, and greater than 75% as significant heterogeneity. Sensitivity analysis was performed by to address moderate to high heterogeneity by excluding outliers.

## Results

**Figure 1:**
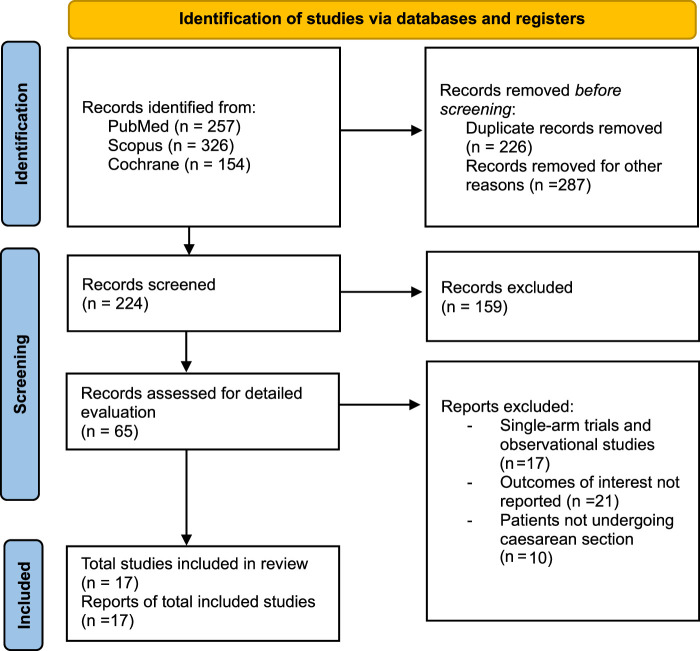
Preferred Reporting Items for Systematic Review and Meta-Analysis (PRISMA) flowchart.

A total of 737 articles were shortlisted from the literature search. After removing duplicates and articles based on study design, patient population, and intervention, a total of 65 were given a full-text evaluation (Fig. 1). A total of 17 studies falling under our eligibility criteria were included in this systematic review and meta-analysis^14–30^. From these 17 studies, 13 had data on pethidine dose less than 20 mg and 11 included doses greater than 20 mg. This cut-off was used due to the greater and lesser effects of pethidine and approximately equal number of studies on both sides of the 20 mg mark. Seven hundred thirty-eight patients were included in the pethidine group and 566 in the control group. Study characteristics are shown in Table 1. Detailed characteristics of the included RCTs are shown in Table 2.

**Table 1:** Characteristics of the included studies

Authors’ names	Year	Country	Dose, mg	Pethidine/control (number)	Shivering (%)
Girma *et al.* ^18^	2022	Ethiopia	10	Pethidine (*n*=43)	9 (20.9)
				Control (*n*=43)	23 (53.5)
Azemati *et al.* ^21^	2022	Iran	10	Pethidine (*n*=30)	1 (3.33)
				Control (*n*=30)	12 (40)
Khezri *et al.* ^17^	2018	Iran	50	Pethidine (*n*=50)	33 (66)
				Control (*n*=50)	41 (82)
Shami *et al.* ^19^	2016	Iran	5	Pethidine (*n*=50)	11 (22)
				Control (*n*=50)	25 (50)
			10	Pethidine (*n*=50)	2 (4)
				Control (*n*=50)	25 (50)
Rastegarian *et al.* ^16^	2013	Iran	25	Pethidine (*n*=50)	4 (8)
				Control (*n*=50)	15 (30)
Yu *et al.* ^15^	2002	China	10	Pethidine (*n*=20)	3 (15)
				Control (*n*=20)	8 (40)
Hirmanpour *et al.* ^14^	2017	Iran	25	Pethidine (*n*=40)	2 (5)
				Control (*n*=40)	26 (65)
Zabetian *et al.* ^29^	2013	Iran	10	Pethidine (*n*=35)	5 (14.2)
				Control (*n*=35)	33 (94.2)
Mahmoud *et al.* ^28^	2016	Egypt	10	Pethidine (*n*=30)	8 (26.6)
				Control (*n*=30)	20 (66.6)
Nasseri *et al.* ^20^	2017	Iran	10	Pethidine (*n*=30)	2 (6.6)
				Control (*n*=30)	18 (60)
Hong *et al.* ^27^	2005	South Korea	10	Pethidine (*n*=30)	1 (3.3)
				Control (*n*=30)	7 (23.3)
Anaraki *et al.* ^26^	2012	Iran	15	Pethidine (*n*=39)	15 (38.4)
				Control (*n*=39)	19 (48.7)
			25	Pethidine (*n*=39)	11 (28.2)
				Control (*n*=39)	19 (48.7)
			30	Pethidine (*n*=39)	6 (15.3)
				Control (*n*=39)	19 (48.7)
Atalay *et al.* ^23^	2010	Turkey	25	Pethidine (*n*=20)	0 (0.0)
				Control (*n*=20)	10 (50)
			30	Pethidine (*n*=20)	0 (0.0)
				Control (*n*=20)	10 (50)
			35	Pethidine (*n*=20)	0 (0.0)
				Control (*n*=20)	10 (50)
Khan *et al.* ^25^	2011	Iran	10	Pethidine (*n*=24)	9 (37.5)
				Control (*n*=24)	18 (75)
			25	Pethidine (*n*=24)	2 (8.3)
				Control (*n*=24)	18 (75)
Shrestha *et al.* ^22^	2007	Nepal	10	Pethidine (*n*=30)	12 (40)
				Control (*n*=30)	23 (76.6)
Roy *et al.* ^24^	2004	Canada	15	Pethidine (*n*=20)	4 (20)
				Control (*n*=20)	15 (75)
Mohamed *et al.* ^13^	2018	Egypt	25	Pethidine (*n*=25)	6 (24)
				Control (*n*=25)	22 (88)

**Table 2:** Detailed trial characteristics

				Mean age		
Authors’ names	Centers	Randomized	Analyzed	Treatment	Control	Trial registration number
Girma *et al.* ^26^	Single	86	86	25.9±3.7	27.2±3.5	PACTR202110617970132
Azemati *et al.* ^29^	Single	60	60	29.3 ± 1.1	30.4 ± 1.1	IRCT2014100814372N4
Khezri *et al.* ^25^	Single	100	100	27.9±6.1	28.3±7.8	IRCT201104073051N4
Shami *et al.* ^27^	Single	150	150	31.3±5.5	31.8±4.7	IRCT2015090223869N1
Rastegarian *et al.* ^24^	Single	100	100	27.0±6.1	26.3±3.7	—
Yu *et al.* ^23^	Single	40	40	33.0±6.0	33.0±5.0	—
Hirmanpour *et al.* ^14^	Multiple	80	80	30.9±5.9	31.1±4.9	—
Zabetian *et al.* ^22^	Multiple	70	70	28.5±7.3	27.1±4.3	IRCT2012090410743N1
Mahmoud *et al.* ^21^	Single	60	60	30.8±3.2	30.4±4.1	—
Nasseri *et al.* ^28^	Single	63	60	29.4±6.0	29.8±5.3	IRCT2013091414656N1
Hong *et al.* ^20^	Single	60	60	30.8±4.3	31.3±4.5	—
Anaraki *et al.* ^19^	Multiple	156	156	28.7±5.0	28.7±5.0	IRCT138904101936N3
Atalay *et al.* ^16^	Single	80	80	28.5±6.1	27.8±4.3	—
Khan *et al.* ^18^	Single	72	72	28.0±6.3	27.1±8.3	—
Shrestha *et al.* ^15^	Single	60	60	—	—	—
Roy *et al.* ^17^	Single	40	40	31±5	32±6	—
Mohamed *et al.* ^13^	Single	50	50	—	—	—

### Meta-analysis

#### Shivering

**Figure 2:**
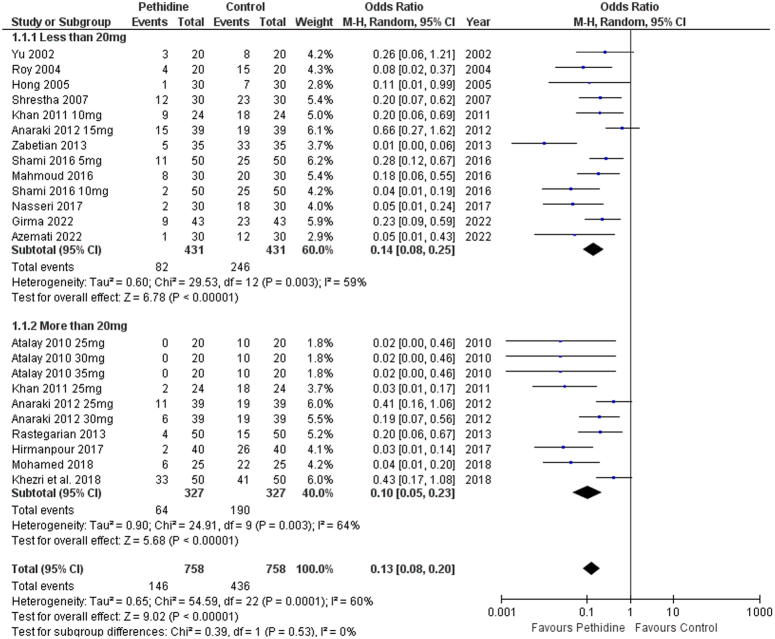
Forest plot of incidence of shivering.

A total of 23 cohorts of pethidine and control were added to the incidence of shivering outcome. Overall, the pethidine group was associated with a decreased incidence of shivering [OR: 0.13 (0.08, 0.20) *P*<0.00001; I^2^=60%]. Pethidine was significantly associated with decreased shivering events post-spinal anesthesia in both, low-dose (<20 mg) and high-dose (>20 mg) pethidine subgroups [OR: 0.14 (0.08, 0.25) *P*<0.00001; I^2^=59%] and [OR: 0.10 (0.05, 0.23) *P*<0.00001; I^2^=64%], respectively (Fig. 2). A sensitivity analysis addressed heterogeneity by removing outliers, which decreased the overall heterogeneity to 48% (Figure S1, Supplemental Digital Content 4, http://links.lww.com/MS9/A566).

#### Intensity of shivering

**Figure 3:**
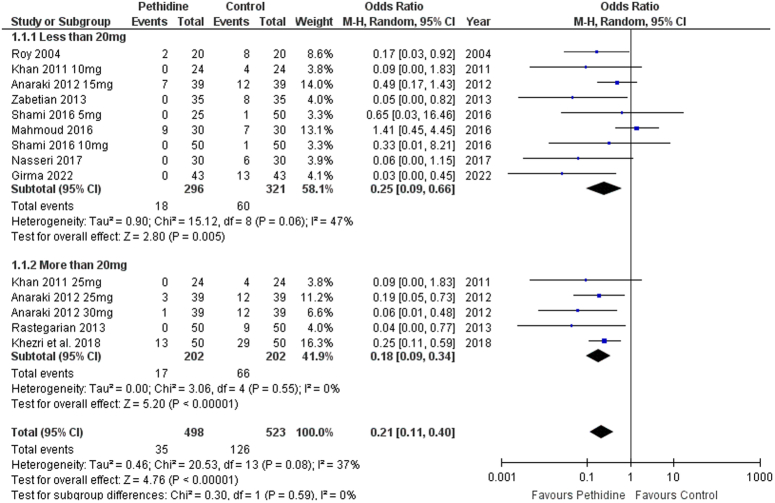
Forest plot of intensity of shivering.

A random-effects meta-analysis was performed to evaluate the association between pethidine and intensity of shivering. Overall, pethidine was associated with a decreased incidence of severe shivering (Stage 3–4) [OR: 0.21 (0.11, 0.40) *P*<0.00001; I^2^=37%]. Low-dose [OR: 0.25 (0.09, 0.66) *P*<0.00001; I^2^=47%] and high-dose [OR: 0.18 (0.09, 0.34) *P*<0.00001; I^2^=0%] pethidine subgroups had similar findings as both were found to be associated with lesser intensity of shivering (Fig. 3).

#### Adverse effects

**Figure 4:**
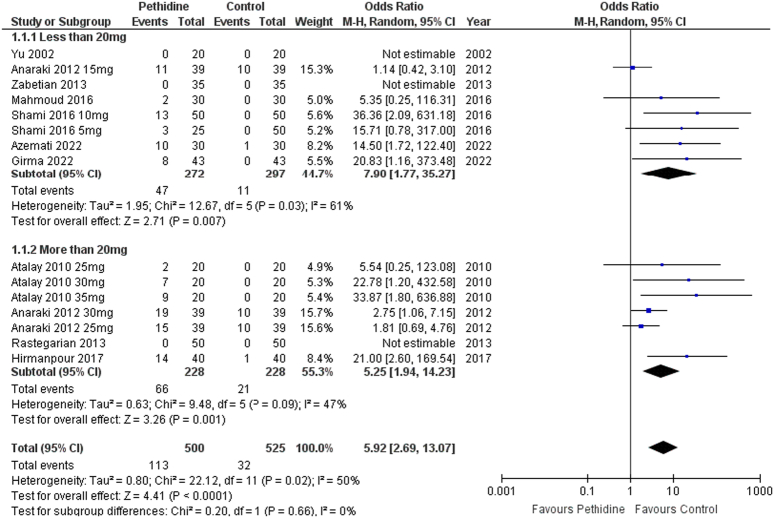
Forest plot of incidence of pruritus.

Incidence of pruritus was evaluated in pethidine and control groups. Pethidine was significantly associated with increased events of pruritus in patients following spinal anesthesia [OR: 5.92 (2.69, 13.07) *P*<0.0001; I^2^=50%]. Both the low-dose [OR: 7.90 (1.77, 35.27) *P*=0.007; I^2^=61%] and high-dose groups [OR: 5.25 (1.94, 14.23) *P*=0.0001; I^2^=47%] had similar findings (Fig. 4).

**Figure 5:**
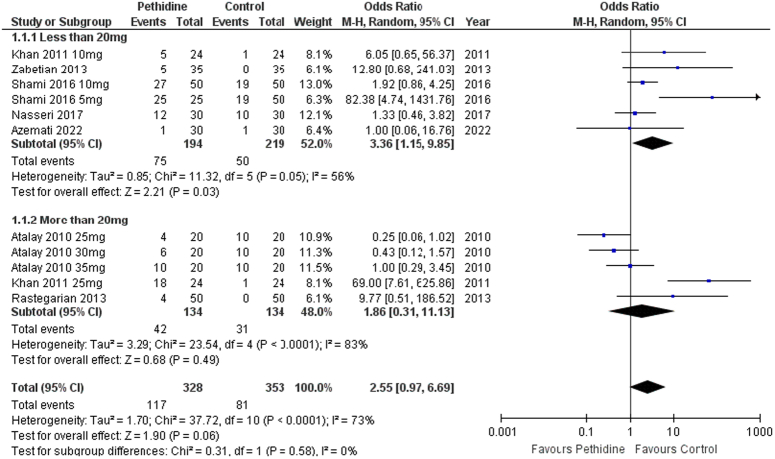
Forest plot of incidence of nausea.

A total of 11 cohorts of pethidine and control evaluated nausea events following spinal anesthesia. Pethidine was non-significantly associated with increased events of pruritus in patients following spinal anesthesia [OR: 2.55 (0.97, 6.69) *P*=0.06; I^2^=73%]. Low-dose pethidine was significantly associated with increased nausea events [OR: 3.36 (1.15, 9.85) *P*=0.03; I^2^=56%]. However, the high-dose group [OR: 1.86 (0.31, 11.13) *P*=0.49; I^2^=83%] had no significant association (Fig. 5).

**Figure 6:**
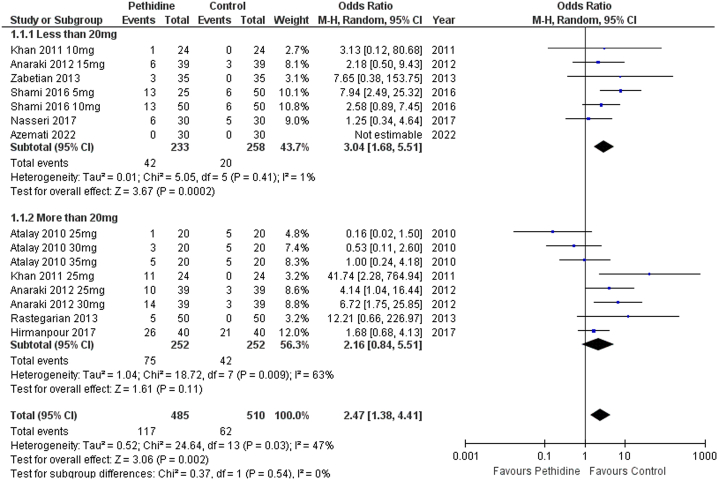
Forest plot of incidence of vomiting.

Pethidine was significantly associated with increased events of vomiting in patients following spinal anesthesia [OR: 2.47 (1.38, 4.41) *P*=0.002; I^2^=47%]. Low-dose pethidine was also significantly associated with increased vomiting in patients following C-section [OR: 3.04 (1.68, 5.51) *P*=0.0002; I^2^=1%]. However, the high-dose group [OR: 2.16 (0.84, 5.51) *P*=0.11; I^2^=63%] had no significant association (Fig. 6).

**Figure 7:**
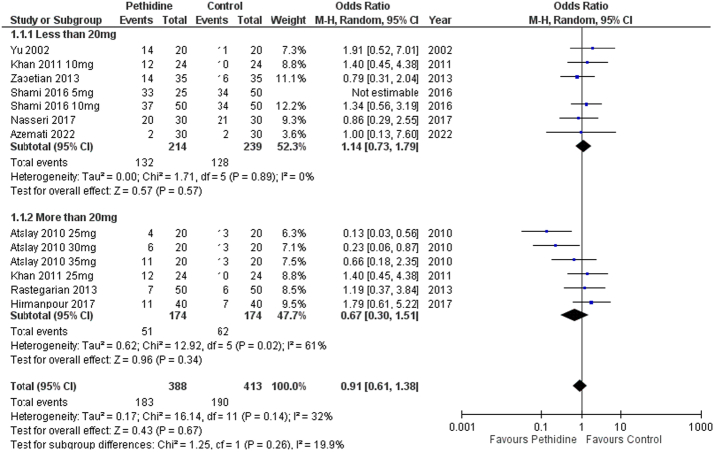
Forest plot of incidence of hypotension.

Hypotension was also evaluated in C-section patients following spinal anesthesia. Overall, no significant association was derived between pethidine and hypotension [OR: 0.91 (0.61, 1.38) *P*=0.67; I^2^=32%]. Both the low-dose [OR: 1.14 (0.73, 1.79) *P*=0.57; I^2^=0%] and high-dose groups [OR: 0.67 (0.30, 0.51) *P*=0.34; I^2^=61%] had similar findings (Fig. 7).

#### Quality assessment and publication bias

Quality assessment was performed using RoB-2 tool in which five domains are assessed for bias. All the included RCTs were found to be at a low risk of bias. Three studies showed some concerns in the randomization process whereas, two studies showed deviation from the intended interventions. Moreover, one study had a moderate risk of missing outcome data. Two articles had some concerns with the measurement of the outcome and three studies had some bias in the selection of results that were discussed. Detailed quality assessment is shown in supplementary figure S2, Supplemental Digital Content 5, http://links.lww.com/MS9/A567. Publication bias was assessed using funnel plot for the primary outcome shivering. Asymmetry was noted upon evaluating the funnel plot (Figure S3, Supplemental Digital Content 6, http://links.lww.com/MS9/A568). This was confirmed by Egger’s regression test showing a two-tailed p-value of 0.0001.

## Discussion

In this meta-analysis we evaluated the impact of low (<20 mg) and high (>20 mg) dose of intrathecal pethidine on the incidence and severity of post-spinal shivering in women undergoing CS. Our study showed that intrathecal administration of pethidine in patients can significantly reduce post-spinal anesthesia shivering events. Additionally, pethidine was linked with lower severity of shivering. The intrathecal pethidine, however, increases the risk of pruritus and vomiting. No association was observed with nausea and hypotension events.

In the majority of the studies in our meta-analysis similar surgical procedures were followed when administering pethidine intrathecally. Firstly, patients received 5–7 ml/kg lactated Ringer’s solution before spinal anesthesia. Then, a 25-gauge Quincke needle was inserted intrathecally via a midline approach between L3/L4 and L4/L5, while the patient was in a sitting position with 2–2.5 ml bupivacaine 0.5% with the prespecified dosage of pethidine.

A meta-analysis conducted by Lin *et al.* ^31^ studied the effect of low-dose intrathecal pethidine in patients undergoing various surgical procedures. They found significantly lower incidence and severity of post-spinal anesthesia shivering; however, nausea and vomiting were increased. Similar findings were observed by Subramani *et al.* ^32^, who found intrathecal pethidine to lower severity and incidence of shivering in CS patients. The results of our meta-analysis are consistent with these findings. Recent randomized controlled trials by Girma *et al.* ^19^ and Azemati *et al.* ^22^. show similar findings as shivering incidence was significantly reduced and severity of shivering was also found to be decreased in patients in the pethidine group.

Spinal anesthesia impedes tonic vasoconstriction and causes readjustment of core heat from the trunk to the periphery thereby patients develop hypothermia and shivering. Post-spinal anesthesia shivering is an involuntary, repetitive activity of skeletal muscles as a reaction to core hypothermia to raise metabolic heat production^9^.

Intravenous pethidine reduces the incidence of post-spinal anesthesia shivering by the reduction of shivering threshold and binding to the kappa receptors^9,33^. Possible mechanisms for intrathecal pethidine to reduce shivering could be k-opioid receptor activity, anticholinergic action, biogenic monoamine reuptake inhibition, NMDA receptor antagonism, or stimulation of alpha 2–adrenoceptors, and possibly modulating the heat loss caused by vasodilatation after spinal anesthesia^34–36^.

In a recent RCT by Azemati *et al.* ^22^ there was no significant association between intrathecal pethidine and nausea and vomiting. Similar findings were observed by Nasseri *et al.* ^21^ These results are contrary to what Lin *et al.* ^31^ found as low-dose intrathecal pethidine increased the incidence of nausea and vomiting. This is consistent with our meta-analysis as the low-dose (<20 mg) subgroup showed increased nausea and vomiting events whereas the high dose (>20 mg) showed no significant association. Shami *et al.* ^20^ found 5, 10, 15 mg of intrathecal pethidine to be of no significance in causing vomiting and nausea. Since pethidine is an opiate, it inhibits the neurotransmission of pain by binding to the opioid receptors in the central nervous system^37^. Thus, pethidine could be attributed to nausea and vomiting caused by stimulation of the medullary chemoreceptor trigger zone^38^.

In our study, there was a significant association between both low- and high-dose intrathecal pethidine and pruritus. These findings are consistent with the study conducted by Jaafarpour *et al.* ^39^. Recent RCT by Azemati *et al.* ^22^ found that concomitant use of pethidine and bupivacaine increased the feeling of itching, which is also consistent with Bi *et al.* ^40^ as they compared the group that received hyperbaric bupivacaine with the groups treated by the pethidine in terms of side effects. They found that itching complications were more prevalent in the pethidine group.

Our meta-analysis is not without its limitations. Firstly, the included RCTs had a low sample size. Secondly, we decided on a low and high-dose cut-off of 20 mg on our own due to the equal statistical distribution of studies on either side and the low and high effects of pethidine observed on either side of the cut-off. Due to a low number of participants in some studies, it contributed to greater CIs in outcomes which resulted in increased heterogeneity. However, our meta-analysis has its strengths also. We included recent RCTs and conducted the meta-analysis with the greatest sample size as compared to previous meta-analysis on this topic. We found crucial dose-dependent associations that were not made previously. Since pethidine was statistically associated with decreasing severity and reduced post-spinal anesthesia shivering events, it could serve as a potent treatment in the clinical setting. However, caution is warranted with the dosing of pethidine as low dose (<20 mg) was associated with increased adverse events.

## Conclusion

Intrathecal pethidine is effective in reducing the severity and incidence of post-spinal anesthesia shivering. However, it increases pruritus, nausea, and vomiting in low doses and it had no association with hypotension.

## Provenance and peer review

Not commissioned, externally peer-reviewed.

## Supplementary Material

## References

[1] Hunt CO Naulty JS Bader AM. Perioperative analgesia with subarachnoid fentanyl-bupivacaine for cesarean delivery. Anesthesiology 1989;71:535–540. 2679237 10.1097/00000542-198910000-00009

[2] Uppal V Retter S Casey M. Efficacy of intrathecal fentanyl for cesarean delivery: a systematic review and meta-analysis of randomized controlled trials with trial sequential analysis. Anesth Analg 2020;130:111–125. 30633056 10.1213/ANE.0000000000003975

[3] Javed S Hamid S Amin DF. Spinal anesthesia induced complications in caesarean section-a review. 2011. J. Pharm. Sci. & Res. 3(10) 2011,1530–1538. https://citeseerx.ist.psu.edu/document?repid=rep1&type=pdf&doi=31e48f77aab9c1f85b1a9d8146d64b21e34c684f

[4] Rollins M Lucero J. Overview of anesthetic considerations for cesarean delivery. Br Med Bull 2012;101:105–125. 22219238 10.1093/bmb/ldr050

[5] Kintu A Abdulla S Lubikire A. Postoperative pain after cesarean section: assessment and management in a tertiary hospital in a low-income country. BMC Health Serv Res 2019;19:1–6. 30683083 10.1186/s12913-019-3911-x PMC6347795

[6] Qi X Chen D Li G. Risk factors associated with intraoperative shivering during caesarean section: a prospective nested case-control study. BMC Anesthesiol 2022;22:1–10. 35227213 10.1186/s12871-022-01596-7 PMC8883627

[7] Javed S Hamid S Amin DF. Spinal anesthesia induced complications in caesarean section-a review. 2011. https://api.semanticscholar.org/CorpusID:15568856

[8] Han JW Kang HS Choi SK. Comparison of the effects of intrathecal fentanyl and meperidine on shivering after cesarean delivery under spinal anesthesia. Korean J Anesthesiol 2007;52:657.

[9] De Witte J Sessler DI. Perioperative shiveringphysiology and pharmacology. Anesthesiology 2002;96:467–484. 11818783 10.1097/00000542-200202000-00036

[10] Page MJ McKenzie JE Bossuyt PM. The PRISMA 2020 statement: an updated guideline for reporting systematic reviews. Int J Surg 2021;88:105906. 33789826 10.1016/j.ijsu.2021.105906

[11] McKenzie JE Salanti G Lewis SC. Meta-analysis and the cochrane collaboration: 20 years of the cochrane statistical methods group. Syst Rev 2013;2:80. 24280020 10.1186/2046-4053-2-80 PMC4219183

[12] Sterne JAC Savović J Page MJ. RoB 2: a revised tool for assessing risk of bias in randomised trials. BMJ 2019;366:l4898. 31462531 10.1136/bmj.l4898

[13] Shea BJ Reeves BC Wells G. AMSTAR 2: a critical appraisal tool for systematic reviews that include randomised or non-randomised studies of healthcare interventions, or both. BMJ 2017;358:4008. 10.1136/bmj.j4008 PMC5833365 28935701

[14] Mohamed G Ahmed N Hameed A. Comparative study between the addition of pethidine Vs fentanyl to hyperbaric bupivacaine for spinal anesthesia in caesarean section. Egypt J Hosp Med 2018;73:5813–5817.

[15] Hirmanpour A Talakoub R Shafa A. Evaluating the effect of intrathecal sufentanil and meperidine on shivering after caesarean section under spinal anesthesia. Arch Anesth Crit Care 2017;3:365–372.

[16] Yu SC Ngan Kee WD Kwan ASK. Addition of meperidine to bupivacaine for spinal anaesthesia for Caesarean section. Br J Anaesth 2002;88:379–383. 11990270 10.1093/bja/88.3.379

[17] Rastegarian A Ghobadifar MA Kargar H. Intrathecal meperidine plus lidocaine for prevention of shivering during cesarean section. Korean J Pain 2013;26:379. 24156005 10.3344/kjp.2013.26.4.379 PMC3800711

[18] Khezri MB Al-sadat Mosallaei M Ebtehaj M. Comparison of preemptive effect of intravenous ketorolac versus meperidine on postoperative shivering and pain in patients undergoing cesarean section under spinal anesthesia: a prospective, randomized, double-blind study. Casp J Intern Med 2018;9:151–157. 10.22088/cjim.9.2.151 PMC5912223 29732033

[19] Girma T Alemu W Assen S. Effect of prophylactic intrathecal pethidine on the incidence of shivering on mothers undergoing cesarean section under spinal anesthesia: a randomized controlled trial. Front Med 2022;9:887724. 10.3389/fmed.2022.887724 PMC9365984 35966870

[20] Shami S Nasseri K Shirmohammadi M. Effect of low dose of intrathecal pethidine on the incidence and intensity of shivering during cesarean section under spinal anesthesia: a randomized, placebo-controlled, double-blind clinical trial. Drug Des Devel Ther 2016;10:3005–3012. 10.2147/DDDT.S115201 PMC5036596 27703328

[21] Nasseri K Ghaderi E Khezripour E. Comparison of the effects of intrathecal meperidine and morphine on incidence and intensity of shivering after caesarean sections under spinal anesthesia: a randomized controlled trial. Iran Red Crescent Med J 2017;19:e55567.

[22] Azemati S Zarghami A Jouybar R. Analgesic characteristics of bupivacaine alone and in combination with dexmedetomidine or meperidine in spinal anesthesia during cesarean section: a double-blind randomized clinical trial study. Pain Res Manag 2022;2022:5111214;. 35899020 10.1155/2022/5111214 PMC9314158

[23] Shrestha BR Maharjan SK Thapa C. Comparative study between bupivacaine heavy vs pethidine intrathecally to study early haemodynamic changes and postoperative analgesia in patients undergoing caesarean section. Kathmandu Univ Med J (KUMJ) 2023;5:166–172. 18604013

[24] Atalay C Aksoy M Aksoy AN. Combining intrathecal bupivacaine and meperidine during caesarean section to prevent spinal anaesthesia-induced hypotension and other side-effects. J Int Med Res 2010;38:1626–1636. 21309476 10.1177/147323001003800507

[25] Roy JD Girard M Drolet P. Intrathecal meperidine decreases shivering during cesarean delivery under spinal anesthesia. Anesth Analg 2004;98:230–234. 14693625 10.1213/01.ANE.0000093251.42341.74

[26] Khan ZH Zanjani AP Makarem J. Antishivering effects of two different doses of intrathecal meperidine in caesarean section: a prospective randomised blinded study. Eur J Anaesthesiol 2011;28:202–206. 21325901 10.1097/EJA.0b013e3283430802

[27] Anaraki AN Mirzaei K. The effect of different intrathecal doses of meperidine on shivering during delivery under spinal anesthesia. Int J Prev Med 2023;3:706. PMC3482998 23112897

[28] Hong JY Lee IH. Comparison of the effects of intrathecal morphine and pethidine on shivering after Caesarean delivery under combined-spinal epidural anaesthesia. Anaesthesia 2005;60:1168–1172. 16288613 10.1111/j.1365-2044.2005.04158.x

[29] Mahmoud MS Kamal MM Abdellatif AM. Effect of intrathecal meperidine and intravenous amino acid infusion in reducing intraoperative shivering during spinal anesthesia: a prospective randomized trial. Egyptian Journal of Anaesthesia 2023;32:391–396. Accessed September 16, 2023. https://www.tandfonline.com/doi/abs/10.1016/j.egja.2016.04.005

[30] Zabetian H Jahromi AS Karami MY. Antishivering effect of low dose meperidine in caesarean section under spinal anesthesia: a randomized double-blind placebo-controlled trial. Int J Pharmacol 2013;9:305–311.

[31] Lin YC Chen CY Liao YM. Preventing shivering with adjuvant low dose intrathecal meperidine: a meta-analysis of randomized controlled trials with trial sequential analysis. Sci Rep 2017;7. doi:10.1038/s41598-017-14917-5 PMC5681692 29127294

[32] Subramani Y Nagappa M Kumar K. Effect of intrathecal lipophilic opioids on the incidence of shivering in women undergoing cesarean delivery after spinal anesthesia: a systematic review and bayesian network meta- analysis of randomized controlled trials. BMC Anesthesiol 2020;20:214. 32847522 10.1186/s12871-020-01116-5 PMC7448354

[33] Lopez MB. Postanaesthetic shivering – from pathophysiology to prevention. Rom J Anaesth Intensive Care 2018;25:73. 29756066 10.21454/rjaic.7518.251.xum PMC5931188

[34] Kurz M Belani KG Sessler DI. Naloxone, meperidine, and shivering. Anesthesiology 1993;79:1193–1201. 8267194 10.1097/00000542-199312000-00009

[35] Ebert B Andersen S Krogsgaard-Larsen P. Ketobemidone, methadone and pethidine are non-competitive N-methyl-d-aspartate (NMDA) antagonists in the rat cortex and spinal cord. Neurosci Lett 1995;187:165–168. 7624018 10.1016/0304-3940(95)11364-3

[36] Larsen JJ Hyttel J. 5-HT-uptake inhibition potentiates antinociception induced by morphine, pethidine, methadone and ketobemidone in rats. Acta Pharmacol Toxicol (Copenh) 1985;57:214–218. 2865865 10.1111/bcpt.1985.57.3.214

[37] Al-Hasani R Bruchas MR. Molecular mechanisms of opioid receptor-dependent signaling and behavior. Anesthesiology 2011;115:1363. 22020140 10.1097/ALN.0b013e318238bba6 PMC3698859

[38] Moon SH. Sedation regimens for gastrointestinal endoscopy. Clin Endosc 2014;47:135. 24765595 10.5946/ce.2014.47.2.135 PMC3994255

[39] Jaafarpour M Taghizadeh Z Shafiei E. The effect of intrathecal meperidine on maternal and newborn outcomes after cesarean section: a systematic review and meta-analysis study. Anesthesiol Pain Med 2020;10:100375. 10.5812/aapm.100375 PMC7322789 32637349

[40] Bi YH Cui XG Zhang RQ. Low dose of dexmedetomidine as an adjuvant to bupivacaine in cesarean surgery provides better intraoperative somato-visceral sensory block characteristics and postoperative analgesia. Oncotarget 2017;8:63587. 28969013 10.18632/oncotarget.18864 PMC5609945

