# Supplementary material for: The effect of intrathecal pethidine on post-spinal anesthesia shivering after cesarean section: a systematic review and meta-analysis
Source: Ann Med Surg (Lond). 2024 Jul 22;86(9):5461–70. doi: 10.1097/MS9.0000000000002354 (PMC11374255; doi:10.1097/MS9.0000000000002354)
Supplement: Supplementary file 2 [file ms9-86-5461-s002.docx]

Table S1. Detailed search string used in each database.

| PubMed | (("Anesthesia, Spinal"[Mesh] OR spinal anesthesia) AND cesarean section OR c-section) AND (pethidine OR meperidine) |
| --- | --- |
| Scopus | ((spinal anesthesia) AND cesarean section OR c-section) AND (pethidine OR meperidine) |
| Cochrane Library | ((spinal anesthesia) AND cesarean section OR c-section) AND (pethidine OR meperidine) |
