# Supplementary figures and images for: The effect of intrathecal pethidine on post-spinal anesthesia shivering after cesarean section: a systematic review and meta-analysis
Source: Ann Med Surg (Lond). 2024 Jul 22;86(9):5461–70. doi: 10.1097/MS9.0000000000002354 (PMC11374255; doi:10.1097/MS9.0000000000002354)

Figure S1. Forest plot of sensitivity analysis of shivering.

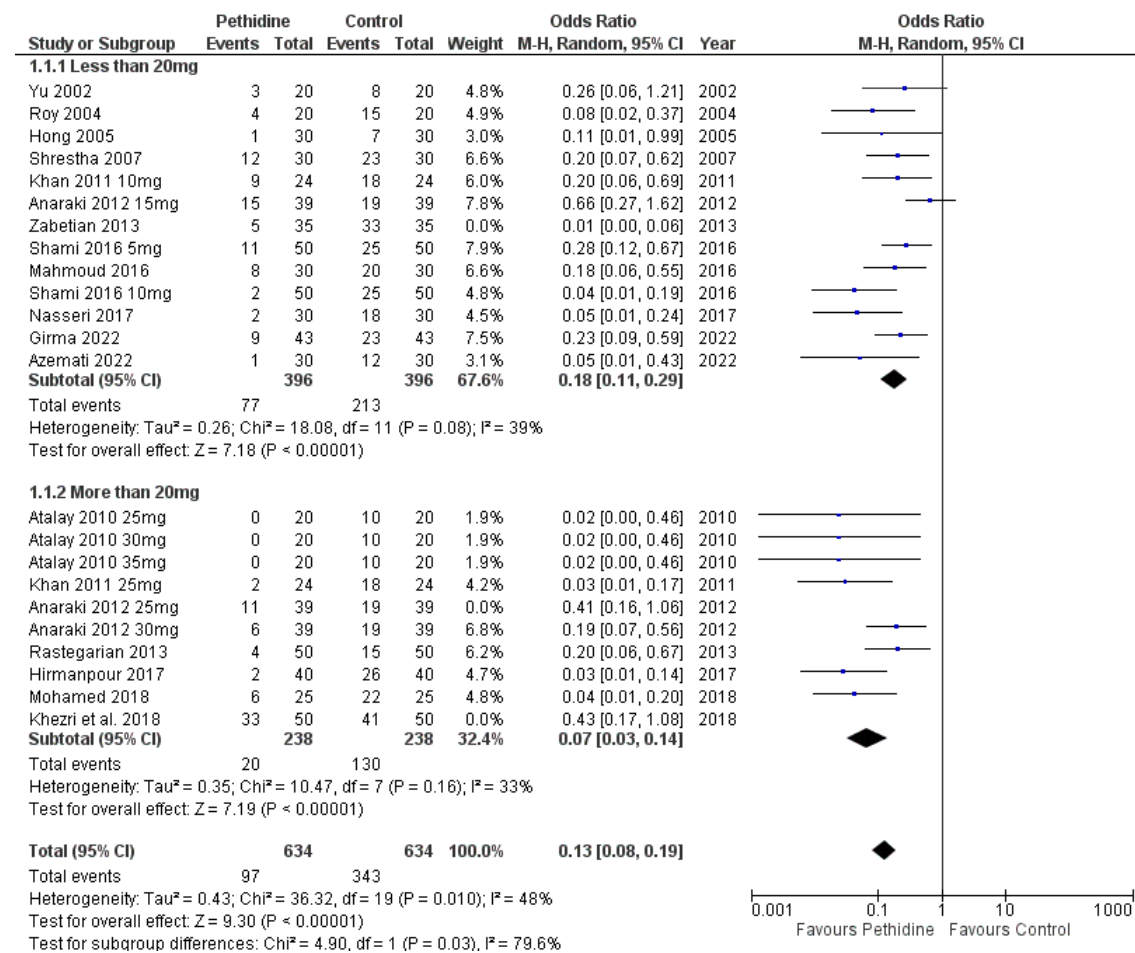

Supplement: Supplementary file 4 [file ms9-86-5461-s004.pdf]

Figure S3. Funnel plot of shivering for publication bias.

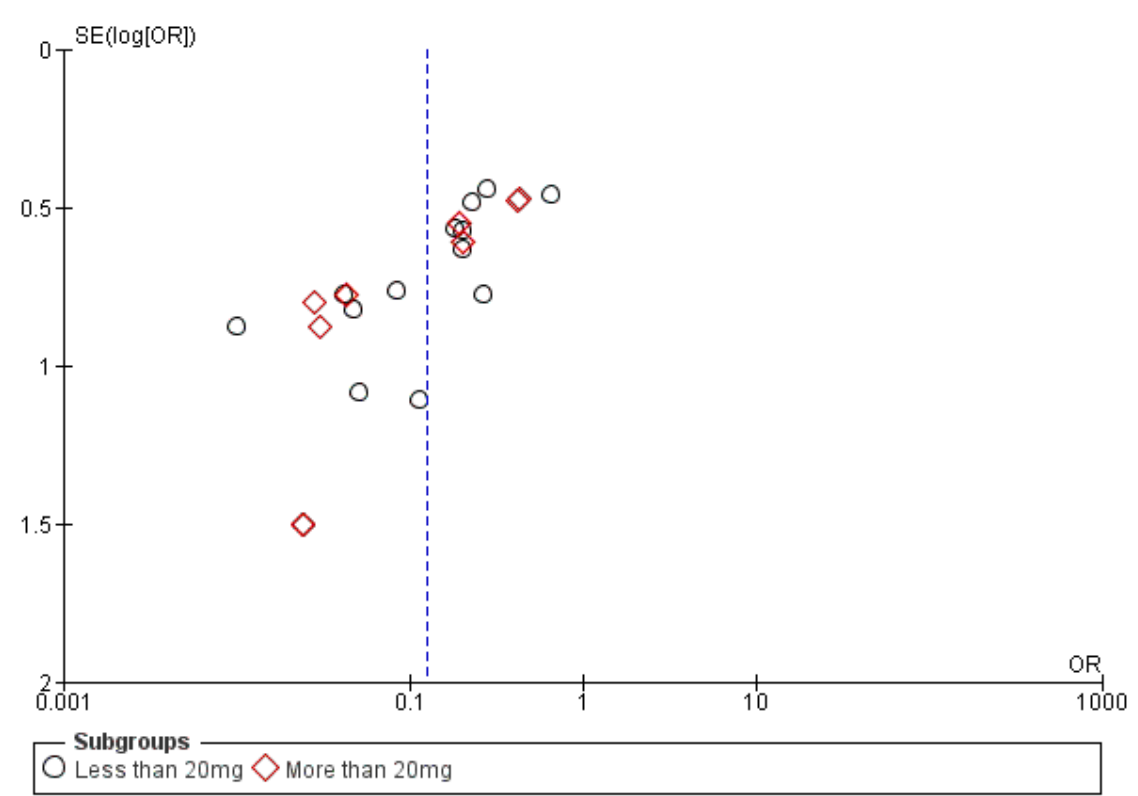

Supplement: Supplementary file 6 [file ms9-86-5461-s006.pdf]
