# Supplementary material for: The effect of intrathecal pethidine on post-spinal anesthesia shivering after cesarean section: a systematic review and meta-analysis
Source: Ann Med Surg (Lond). 2024 Jul 22;86(9):5461–70. doi: 10.1097/MS9.0000000000002354 (PMC11374255; doi:10.1097/MS9.0000000000002354)
Supplement: Supplementary file 5 [file ms9-86-5461-s005.pdf]

Figure S2. Quality assessment of the included studies.

Study Name, Year	Experimental	Comparator	Weight	D1	D2	D3	D4	D5	Overall
Girma 2022	Pethidine	Control	1	+	+	+	+	+	+
Azemati 2022	Pethidine	Control	1	!	+	+	+	+	+
Khezri 2018	Pethidine	Control	1	+	+	+	+	!	+
Shami 2016	Pethidine	Control	1	+	+	!	+	+	+
Hirmanpour 2017	Pethidine	Control	1	+	+	+	+	+	+
Rastegarian 2013	Pethidine	Control	1	+	+	+	!	+	+
Yu 2002	Pethidine	Control	1	+	+	+	+	+	+
Zabetian 2013	Pethidine	Control	1	!	+	+	+	+	+
Mahmoud 2016	Pethidine	Control	1	+	+	+	+	!	+
Nasseri 2017	Pethidine	Control	1	+	!	+	+	+	+
Hong 2005	Pethidine	Control	1	+	+	+	+	+	+
Anaraki 2012	Pethidine	Control	1	+	+	+	!	+	+
Khan 2011	Pethidine	Control	1	+	+	+	+	+	+
Shrestha 2007	Pethidine	Control	1	!	+	+	+	+	+
Roy 2004	Pethidine	Control	1	+	+	+	+	!	+
Mohamed 2018	Pethidine	Control	1	+	!	+	+	+	+
Atalay 2010	Pethidine	Control	1	+	+	+	+	+	+

+	Low risk		
!	Some concerns	D1	Randomisation process
-	High risk	D2	Deviations from the intended interventions
		D3	Missing outcome data
		D4	Measurement of the outcome
		D5	Selection of the reported result
